# A successful periodontal‐orthodontic treatment of an advanced case of dental trauma in a 9‐year‐old patient: A case report with 16‐year follow‐up

**DOI:** 10.1002/cap.70047

**Published:** 2026-02-28

**Authors:** João Carnio, Maísa Casarin, João Kreling Carnio, Flavia Q. Pirih

**Affiliations:** ^1^ Private Practice Londrina Brazil; ^2^ Department of Periodontics, School of Dentistry Federal University of Pelotas (UFPel) Pelotas Brazil; ^3^ School of Dentistry University of Cesumar (UniCesumar) Maringá Brazil; ^4^ Section of Periodontics UCLA School of Dentistry, Los Angeles (UCLA) Los Angeles California USA

**Keywords:** case reports, inflammation, orthodontics, periodontal diseases, tooth injuries, wounds and injuries

## Abstract

**Background:**

Traumatic dental injury (TDI) most often requires complex and interdisciplinary management. Several factors are associated with the success of TDI treatment, including proper treatment planning and periodontal maintenance.

**Methods:**

The present 16‐year follow‐up report describes the interdisciplinary management of an extrusive luxation of teeth #8 and #9 in a 9‐year‐old‐patient.

**Results:**

After the extrusion, endodontic treatment was performed on teeth #8 and #9 and then periodontal treatment with supra and subgingival debridement associated with locally antibiotic application was done. Once periodontal treatment was stable, orthodontic treatment started. During orthodontic treatment, the patient was advised to have periodontal maintenance every 3–4 months. At the 16‐year follow‐up appointment, both teeth were in function and in acceptable esthetic condition.

**Conclusion:**

This report demonstrates that proper diagnosis combined with interdisciplinary therapeutic approaches and periodontal maintenance could lead to long‐term successful and stable outcomes.

**Key points:**

- The effective management and proper treatment of dental trauma depend on an accurate diagnosis.
- Interdisciplinary management is essential for successful treatment of dental trauma.
- Patient adherence to periodontal maintenance directly influences outcomes.

**Plain language summary:**

When dental injuries are diagnosed correctly and treated with the help of different dental specialists—along with regular periodontal care—the long‐term results can be stable and successful.

## INTRODUCTION

Traumatic dental injury (TDI) is a frequent public health concern^1^, ^2^ that has a higher occurrence in children and adolescents, comprising 5% of all injuries.^3^ Almost one third of the population has experienced some type of dental trauma such as tooth, bone, and soft tissue injuries,^1^ with a substantial number of dental‐related emergencies.^4^ The prevalence of the extrusion of permanent teeth is seen between 2% and 3% of all dental injuries.^5^, ^6^ It mostly affects males, and permanent maxillary central incisors.^4^

When a TDI occurs, pain and discomfort can lead to disability specially in children and teenagers.^7^ When it involves the maxillary anterior sextant, it can cause several psychological and functional problems, such as speech impairment, ineffective mastication, esthetic problems,^7^, ^8^ loss of self‐confidence, concern about appearance, and feeling poor bereavement.^9^

To appropriately treat the patients with a TDI, emergency assessment and multidisciplinary treatment planning may be necessary.^1^ In case of severe trauma to permanent teeth, a more complex treatment plan might be needed, and the predictability of the treatment depends on the extent of injury to the teeth and to their supporting structures.^10^ Depending on the TDI, several treatment options should be considered: restoration (with or without pulp capping or partial pulpotomy in case of pulp exposure), gingivectomy, crown lengthening, orthodontic intrusion, repositioning and splinting, replantation, or extraction.^11^

Extrusive luxation injuries are caused by the application of an oblique force, leading to heightened mobility and partial displacement of the tooth from its socket in an incisal/axial direction.^12^, ^13^ Commonly referred to as partial avulsions,^6^, ^14^ these injuries can interrupt the vascular supply to the pulp.^14^, ^15^ Clinically, the tooth exhibits elongated and increased mobility^6^, ^13^; occlusion and mastication can be painful, with any spontaneous pain being generally mild.^16^ Radiographically, the apical and lateral regions of the socket appear empty,^13^ and overall, the periodontal ligament space is enlarged.^6^, ^12^ Prognosis for extrusive permanent teeth depends on the extent and severity of the injury, as well as the viability of the periodontal ligament, root maturity, patient age, and how early the trauma was managed, influencing the treatment and prognosis.^17^ Considering the adverse effects of tooth loss on functional, social, and psychological well‐being, different treatment options should be considered based on the type and extension of the trauma. Besides, when a periodontal condition coexists with traumatic injury, periodontal treatment should be performed. Moreover, patient compliance with regular periodontal maintenance is essential for a favorable long‐term outcome. Therefore, the purpose of this case report is to describe a successful long‐term treatment in an unusual case of extrusive luxation with root reabsorption and periodontal problem of maxillary anterior teeth in a 9‐year‐old patient, with 16‐year follow‐up.

## MATERIALS AND METHODS

**FIGURE 1:**
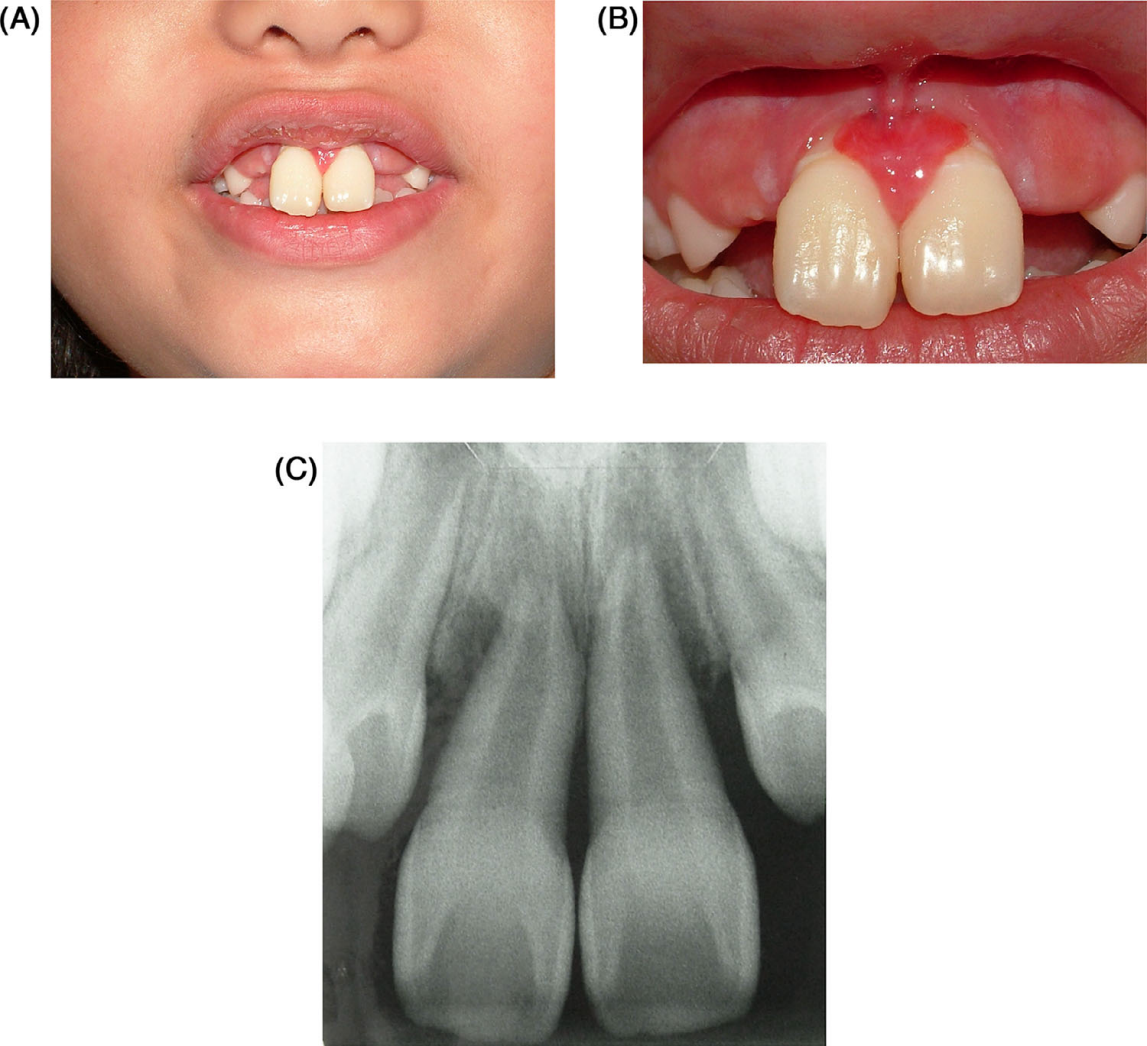
(A) Clinical image from the smile at baseline. (B) Clinical image at baseline. (C) Radiograph image at baseline.

The present case was reported in accordance with the CAse REports(CARE) guidelines.^18^ A 9‐year‐old girl accompanied by her mother was first evaluated in March 2006 at JC's private practice due to a TDI in the permanent maxillary central incisors. The patient was systemically healthy and was not taking any medications. Clinical assessment (Figure 1A,B) and radiographic examination revealed extrusive luxation of teeth #8 and #9 with localized severe gingival inflammation, grade II mobility,^19^ and a vital pulp on both teeth. Based on the clinical and radiographic findings, no endodontic involvement was detected, apical root formation was almost completed, and the radiographic assessment suggested periodontal bone loss of ≥50% involvement in the traumatic teeth (Figure 1C). Patient revealed no pain at that time. The periodontal diagnosis for the patient, excluding teeth #8 and #9, was generalized gingivitis associated with dental plaque biofilm, whereas teeth #8 and #9 were diagnosis with extrusive luxation. Written informed consent was obtained from the patient’s legal guardian.

### Periodontal treatment

The initial phase of treatment plan included the elimination of the gingival inflammation through non‐surgical treatment, short‐term stabilization, periodontal maintenance for 3 months and re‐evaluation of the condition. The patient did not pursue treatment and only returned to the periodontal office 4 years after the trauma, upon referral from the orthodontist. The orthodontist requested a comprehensive evaluation, which included the periodontal prognosis for both maxillary central incisors and periodontal clearance prior to initiating orthodontic treatment.

**FIGURE 2:**
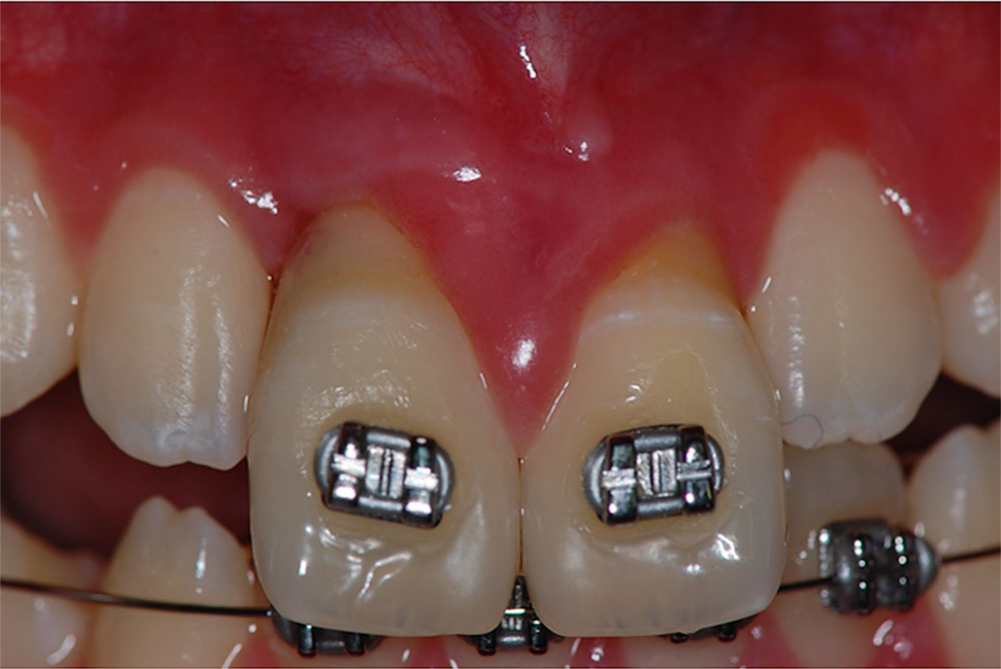
Clinical assessment after 4 years.

**FIGURE 3:**
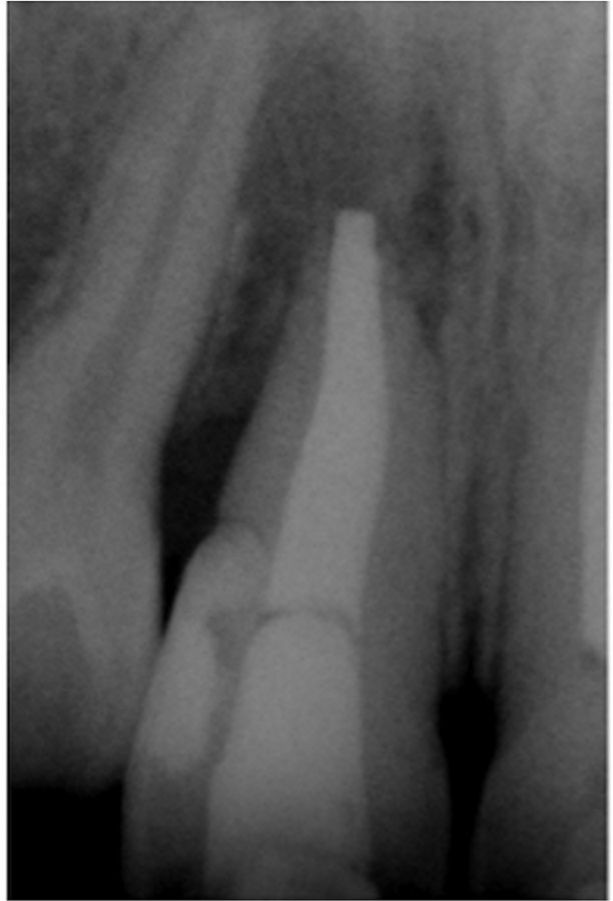
Radiograph image of tooth #8 with external root reabsorption.

Clinical examination revealed that teeth #8 and #9 were extruded and buccally positioned. In addition, gingivitis, gingival recession, and mobility grade II were identified (Figure 2). In the case presented herein, given the young age of the patient, endodontic treatment was only performed once tooth #8 showed clinical and radiographic signs of external root reabsorption. At that time, both teeth were endodontic treated, and the root resorption on tooth #8 was treated with calcium hydroxide (Biodinâmica) and later replaced with resin‐modified glass ionomer cement (3M; Sumaré; Figure 3). Periodontal evaluation revealed ≥5‐mm probing depth on the distobuccal site of tooth #8 and 4‐mm probing depth on the mesiobuccal site (Table 1). Although at this time, the prognosis of teeth #8 and #9 was deemed hopeless,^20^ the intent was to maintain these teeth for as long as possible to preserve the space, as the patient was in the growth phase and was too young for implant placement.

**TABLE 1:** Probing depth and gingival recession in buccal and palatal side (April 2010).

			Tooth #8			
	Distobuccal	Mid‐buccal	Mesiobuccal	Distopalatal	Mid‐palatal	Mesiopalatal
PD	9	5	2	7	2	2
GR	4	4	0	0	0	0
Mobility	Grade II					

			Tooth #9			
PD	4	1	3	2	1	1
GR	3	2	0	0	0	0
Mobility	Grade II					

Abbreviations: GR, gingival recession; PD, probing depth.

Given the intent to preserve the teeth, supragingival scaling, root planing, and subgingival application of 10% doxycycline hydrochloride 10% (Atridox) were performed on teeth #8 and #9 during the consultation to reduce subgingival inflammation. The same protocol was repeated at 3‐month intervals over a period of 9 months.

The use of subgingival doxycycline during the early phase was intended to avoid the need for subgingival scaling and root planing, preventing any damage to or removal of subgingival fibers, as the teeth were extruded, mobile, and exhibited subgingival inflammation.

**FIGURE 4:**
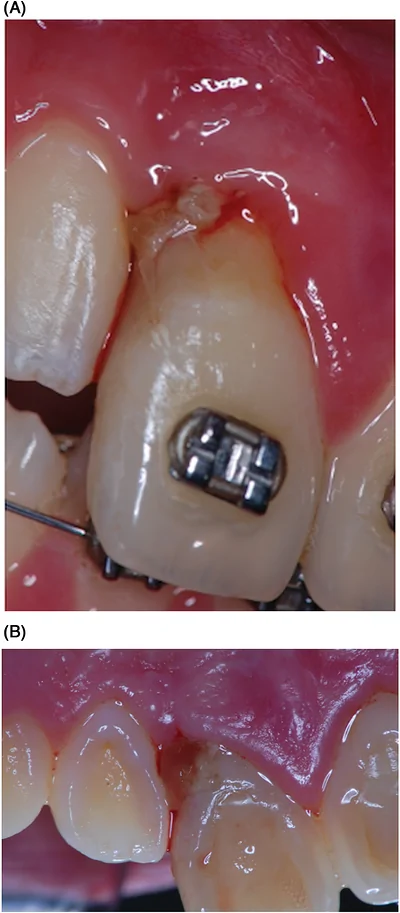
(A) Clinical condition tooth #8 after first subgingival irrigation. (B) Palatal condition tooth #8.

At 9‐month reevaluation, the periodontal treatment plan included subgingival debridement on distobuccal, mid‐buccal, and distopalatal sites (Figure 4A,B).

### Orthodontic therapy

**FIGURE 5:**
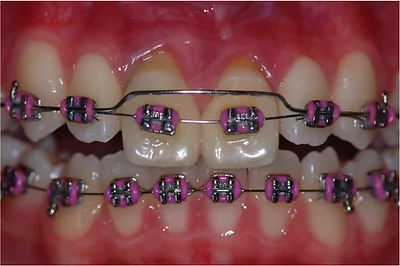
Clinical condition during the orthodontic treatment.

After the patient finished the active periodontal non‐surgical phase, and the disease was controlled, orthodontic treatment was initiated. The importance of adhering to a 3‐ to 4‐month periodontal maintenance interval was emphasized, and the parents agreed with the proposed treatment plan. For facial and orthodontic treatment, vertical growth control forces were performed using an expander with a stabilizer plate. Adjustments were made to the stabilizer plate to promote favorable growth in the vertical and anteroposterior directions, without limiting the transverse direction (Figure 5).

Since the occlusion was concentrated in the anterior teeth region, due to the extensive extrusion, the use of the plate aimed to protect the anterior teeth from premature dental occlusion. Vertical growth control movements were performed; 10‐g intrusive forces and lingual torque forces (without the presence of a buccal bone plate) were utilized. Lower right premolar (#28) extraction was performed, given the bilateral asymmetry, giving preference to canine guide in relation to molars.

During the 11 years of active orthodontic treatment, given the complexity of the case and the patient's limited compliance with the treatment, the patient received at least three periodontal maintenance treatments a year. During these periods, supra and subgingival instrumentation were performed with mini‐five curettes (Hu‐Friedy) and slim ultrasonic subgingival tip (Hu‐Friedy Mfg Co Inc, SATELEC). For supra gingival plaque control, rubber cup polishing and prophy‐jet were utilized. In addition, oral hygiene instructions were carried out according to the needs presented by the patient at the time of the appointment.

### Periodontal and orthodontic maintenance

**FIGURE 6:**
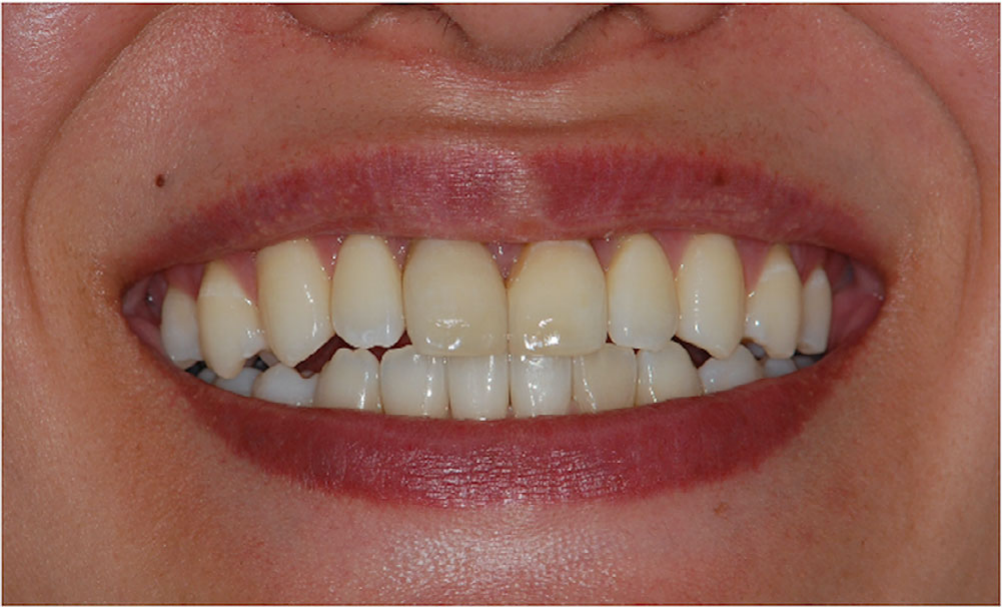
Clinical image after ending orthodontic treatment.

Once the active orthodontic treatment phase was finalized (Figure 6), the patient was given a removable maxillary retainer, a fixed mandibular retainer, and a Michigan stabilizing plate for parafunction control and three‐dimensional stability of the teeth, especially vertical stability. Moreover, the patient continued with the same periodontal maintenance regimen (Table 2). No adverse effects were reported by the patience during the treatment.

**TABLE 2:** Probing depth and gingival recession in buccal and palatal side (February 2023).

			Tooth #8			
	Distobuccal	Mid‐buccal	Mesiobuccal	Distopalatal	Mid‐palatal	Mesiopalatal
PD	6	3	3	3	3	3
GR	1.5	1	0	0	0	0
Mobility	Grade II					

			Tooth #9			
PD	4	2	2	3	2	2
GR	0	1.5	0.5	0	0	0
Mobility	Grade II					

Abbreviations: GR, gingival recession; PD, probing depth.

## RESULTS

**FIGURE 7:**
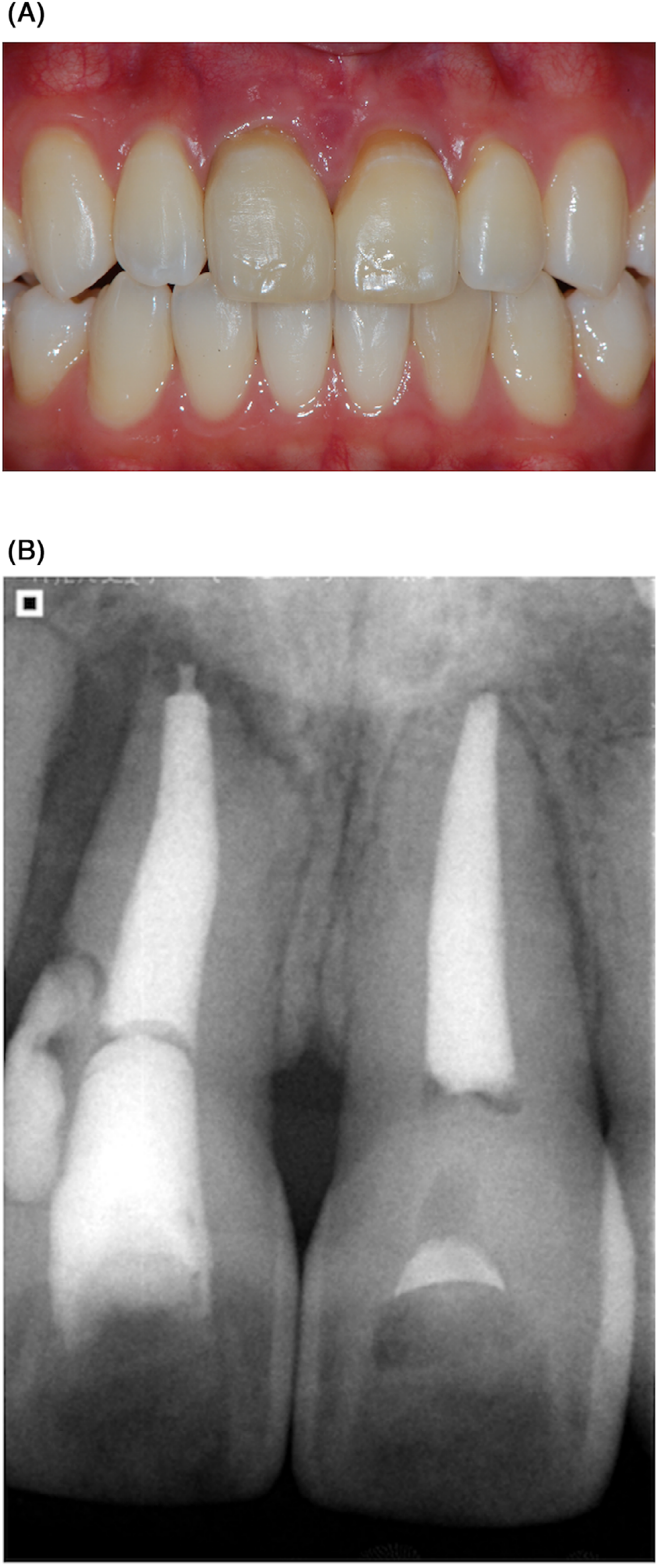
(A) Clinical image after 16 years. (B) Radiograph image after 16 years.

Although the patient did not follow the proposed initial treatment plan and started periodontal treatment only after 4 years of the initial consultation, 16‐year results were still favorable, with both teeth in function and with acceptable esthetics after interdisciplinary approach (periodontal, endodontics, orthodontics, and restorative treatment). Furthermore, clinical parameters improved after the periodontal treatment and orthodontic treatment (Figure 7A) with the absence of residual probing depth >6 mm. Probing depth reduction and clinical attachment gain improved continuously at the 16‐year follow‐up, with a stable mean probing depth of 3 mm. Besides, no bleeding on probing, gingival inflammation, and dental plaque was recorded, denoting a behavioral change with a high‐level of oral hygiene by the patient. The radiographic assessment (Figure 7B) demonstrated stable long‐term results.

## DISCUSSION

When a TDI occurs, proper diagnosis and management are essential in such cases. Notably, the patient was still in the growth phase, with the maxillary and mandibular development ongoing, making implant placement contraindicated.^21^, ^22^ Given the severity of the extrusive luxation and the young age of the patient when the TDI occurred, the multidisciplinary treatment allowed for a successful treatment.

In young patients, it is important to maintain teeth, whenever possible because removing permanent teeth in children and adolescents will influence facial growth.^1^ ^,^ ^23^ For instance, the area, where implants are placed, do not follow the formation and development of the alveolar bone process, which can be seen clinically and radiographically.^24^ The anterior maxilla is the riskiest site for implant placement given that the amount and direction of growth are unpredictable in adolescents.^20^ Some strategies have been attempted to mitigate this risk. For example, the use of transitional implants has been proposed as an alternative in adolescents with a regular follow‐up.^25^ Bonfante et al., in a retrospective case series with 5‐ to 15‐year follow‐up, reported that unsplinted implant‐supported single crowns placed in adolescents exhibited high survival rates, with the most common complications being loss of proximal contacts and infraocclusion.^26^ Despite these findings, delaying implant placement until skeletal growth is complete remains the most appropriate strategy.^21^ Implant placement in children and adolescents may lead to unesthetic and non‐functional outcomes, such as infraocclusion and periodontal complications, and therefore should be indicated with caution.^21^, ^22^

In immature permanent teeth, maintaining tooth vitality is of utmost importance to allow continued root development and apex formation. Moreover, the pulp of an immature permanent teeth has capacity for healing after a traumatic pulp exposure, luxation injury, or even root fracture. In these cases, monitoring the pulp condition with pulp vitality tests at follow‐up appointments is recommended.^13^ In the case presented herein, given the young age of the patient, pulp vitality was maintained until tooth #8 showed clinical and radiographic signs of external reabsorption. At that time, endodontic treatment was performed. A retrospective longitudinal study involving adolescents reported a 2.3% risk of root resorption following dental trauma.^27^ Root resorption after dental trauma in the permanent dentition continues to be a challenge to the dental practitioner, due to multifactorial etiology, diverse clinical appearance, and the questionable prognosis.^28^

Considering the diagnosis and the treatment plan, the use of complementary examinations is essential for clinical decision‐making, particularly in cases involving dental trauma. A study evaluating images of patients who underwent both periapical radiography and cone‐beam computed tomography (CBCT) simultaneously due to dental trauma in the anterior maxillary dentoalveolar region in children and adolescents demonstrated that CBCT was significantly superior to periapical radiography for the diagnosis of root fractures, alveolar fractures, luxations, and tooth resorption. However, no significant differences were found for the diagnosis of crown fractures and periapical radiolucencies.^29^ Another study compared periapical radiographs and upper standard occlusal radiographs with CBCT in the diagnosis and management of TDI. CBCT imaging was significantly more accurate for all diagnoses of TDI compared to the periapical radiographs.^30^ Therefore, a meticulous case history and clinical examination are indispensable before the CBCT examination.^31^ Although CBCT doses and radiation risk remain in the lower levels^32^ dental CBCT examinations in children, instead of conventional radiography, should be fully justified.^33^

Studies showed that TDIs, which require treatment, negatively affect the quality of life in the children and adolescents.^34^, ^35^ When TDIs affect the anterior sextant, it can have an even more significant psychological, esthetic, and social impact. These injuries can produce significant emotional and social burdens not only for the child but also for the family.^36^ It is important to note that the current treatment plan for extrusive luxation of permanent teeth involves several criteria, such as repositioning the tooth by gently pushing it back into the socket under local anesthesia; stabilizing the tooth for 2 weeks using a passive, flexible splint; monitoring the pulp condition; and performing appropriate endodontic treatment if the pulp becomes necrotic and infected.^13^

Considering that there is communication between the periodontal tissue and the dental pulp through pathways such as lateral and accessory canals, apical foramina, or exposed dentine tubules, the initial attempt was to only perform local application with doxycycline hydrochloride 10% (Atridox), causing minimal disruption, therefore allowing healing in the local area.^37^ There is limited evidence for the use of systemically and locally delivered antibiotics in the management of the TDIs.^13^, ^38^ However, some studies showed that locally delivered doxycycline improves clinical parameters when associated with scaling root planing in patients with periodontitis.^39^, ^40^ Moreover, the guideline for the treatment of periodontitis stage I–III has shown that local delivery antibiotics/antimicrobials as adjunctive treatment to subgingival instrumentation may be considered.^41^ Herein, the use of subgingival doxycycline hydrochloride 10%, despite the tooth having sustained a TDI, may have improved the inflammatory parameters.

It is important to note that TDI care is vital for young people. It is essential to comprehend that the treatment of this type of injury in young patients is often complicated, unpredictable, and extensive and can continue for the remainder of their life.^42^ Instructions regarding the care of the injured area should be performed to allow for better healing and prevent further injury. Appropriate follow‐up is essential to detect and appropriately manage any post‐traumatic complication.^43^, ^44^ Long‐term follow‐up revealed that the resorption of the dental structure has not yet stabilized, indicating that additional intervention may be necessary, potentially involving the use of an alternative restorative material.

The chosen material of the restorative treatment of the resorption in the case report was resin‐modified glass ionomer cement. Abbott and Lin developed a clinical classification system for tooth resorption.^45^ This guideline also addresses considerations related to treatment; however, it does not provide recommendations regarding the choice of restorative materials for resorption defects. The number of original research studies on resorption remains limited. Calcium‐silicate–based materials have been suggested for the management of external cervical resorption defects due to their biocompatibility, antibacterial properties, favorable esthetic outcomes, and excellent sealing ability.^46^ The use of Biodentine (Septodont), followed by glass ionomer and/or composite resin restorations, in cases in which esthetic expectations are high has been recommended.^47^ MTA BIOREP (Itena) is a high‐plasticity, endodontic, bioceramic restorative cement that has also been proposed for such defects.^48^, ^49^ As the clinical case presented resorption involving the crown, cervical region, and root, the selection of an appropriate restorative material becomes even more challenging.

It is also important to remember that orthodontic therapy should not be implemented before controlling periodontal inflammation. In the presence of periodontal inflammation, orthodontic movement can lead to uncontrolled periodontal breakdown.^50^, ^51^ A systematic review concluded that in periodontally stable patients, orthodontic treatment has minimal or no additional loss of attachment.^52^

Periodontal maintenance is characterized as a combination of preventive and therapeutic interventions at different intervals which should include oral hygiene instructions, assessment and monitoring of systemic and periodontal health, patient motivation toward continuous risk factor control, professional mechanical plaque removal, and localized subgingival instrumentation if necessary.^41^ Studies have shown that periodontal treatment combined with adherence to recommended maintenance scheduled and self‐performed plaque control are effective in preserving teeth in function over time.^53^, ^54^ This report shows that periodontal treatment associated with periodontal maintenance could lead to maintainable outcomes.

This case report has some limitation that must be addressed. Despite orthodontic treatment, there is still attachment loss at the distobuccal line angle of tooth #8, which could compromise the esthetic outcome of a future installation of implant supported crown. In addition, the most recent radiographs reveal a radiolucency between the restorative material and the tooth structure, suggesting that the resorption may not be stable. The prognosis of teeth affected by root resorption remains challenging. Nevertheless, the patient was advised regarding the need for continued follow‐up and the possibility of additional intervention.

## CONCLUSION

The successful management of this TDI was achieved through a multidisciplinary approach, with the reestablishment of periodontal health, endodontic treatment, and orthodontic therapy, and the results were maintained for the long term given the strict periodontal maintenance care, which has been carried out over the years.

## AUTHOR CONTRIBUTIONS

**João Carnio**: Conceptualization; funding acquisition; investigation; visualization; writing—review and editing. **Maísa Casarin**: Conceptualization; methodology; investigation; writing—original draft; writing—review and editing. **João Kreling Carnio**: Conceptualization; methodology; writing—review and editing. **Flavia Queiroz Pirih**: Conceptualization; project administration; investigation; writing—review and editing.

## CONFLICT OF INTEREST STATEMENT

The authors declare no conflicts of interest.
